# Load-specific post-activation performance enhancement from back squats in male collegiate basketball players: moderate loads preferentially enhance jump performance and lower-limb EMG activation

**DOI:** 10.3389/fphys.2026.1847856

**Published:** 2026-08-05

**Authors:** Zhan Gao

**Affiliations:** Institute of Physical Education and Training, Capital University of Physical Education and Sports, Beijing, China

**Keywords:** back squat, basketball, electromyography (EMG), jumping performance, post-activation performance enhancement (PAPE)

## Abstract

**Purpose:**

To compare the acute effects of pre-activation with different loads of back squat on multiple jumping performances and lower limb electromyographic (EMG) activity in male collegiate basketball players, and to provide evidence for load selection and timing of pre-game warm-up activation.

**Methods:**

In a randomized, parallel-group controlled design, 40 male collegiate basketball players meeting inclusion criteria were assigned to 70%, 80%, 90% 1RM squat groups and a control group. Each experimental group performed 3 sets × 4 repetitions of parallel back squat as a conditioning activity (CA), while the control group rested quietly. Countermovement jump (CMJ), squat jump (SJ), approach vertical jump (AVJ), and standing long jump (SLJ) were assessed before intervention and at 4, 8, and 12 min post-intervention. Ground reaction force and surface EMG of 8 muscles on the dominant lower limb were recorded simultaneously during CMJ and SJ. Jump height/distance, reactive strength index (RSI), elastic utilization ratio (EUR), and EMG root mean square (RMS) were calculated. A 4 (group) × 4 (time point) repeated-measures ANOVA was applied.

**Results:**

Jumping performance showed significant main effects of time under different squat loads, with peak post-activation performance enhancement (PAPE) mainly occurring at 8–12 min post-CA. Compared with 90% 1RM, the 70%–80% 1RM groups exhibited more stable improvements in CMJ, SJ, AVJ, and RSI, along with more consistent within-muscle increases in normalized RMS for rectus femoris (RF), biceps femoris (BF), and lateral gastrocnemius (LG), with minor changes in EUR.

**Conclusion:**

Moderate-load back squat CA induces marked PAPE in male collegiate basketball players within a short time window, with 8–12 min being the optimal period for performance enhancement. Within the load range of this study, moderate-to-high intensity squat appears more effective than high-intensity conditions in acutely improving jumping performance and enhancing within-muscle normalized EMG responses in selected lower-limb muscles. Descriptively, considering static and reactive jump indices and EMG outcomes together, 80% 1RM showed a favorable overall response pattern in this sample, without implying direct statistical superiority over 70% 1RM These findings provide a reference for optimizing basketball-specific warm-up activation programs.

## Introduction

Basketball is an intermittent, confrontational sport characterized by repeated high-intensity shuttle running, sudden stops and starts, directional changes, and vertical jumps (Song and Deng, 2023; Baena-Raya et al., 2024; Cao et al., 2025). It demands high levels of force output and power generation within extremely brief time windows. Lower-limb explosive power and its rapid mobilization are core physical qualities underlying key tactical and technical movements such as rebounding, blocking, driving to the basket, tip-ins, and repeated jumps (Townsend et al., 2019; Pechlivanos et al., 2024). Vertical explosive power is commonly evaluated using countermovement jump (CMJ) and squat jump (SJ), while horizontal explosive power is assessed via standing long jump (SLJ). These tests reflect maximal or near-maximal force output under different muscle contraction modes and, to some extent, indicate stretch-shortening cycle (SSC) utilization and neuromuscular coordination (Markovic et al., 2004; Chen et al., 2023; Marin‐Jimenez et al., 2024). Improving these jump performances acutely through rational training and pre-game warm-up to gain a transient competitive edge has become a key topic in strength and conditioning research.

In recent years, post-activation performance enhancement (PAPE) has emerged as a prominent acute training adaptation (Olsen et al., 2025). PAPE refers to the transient improvement in explosive performances such as jumping and sprinting observed several to ten minutes after a properly designed conditioning activity (CA) (Liu et al., 2024; Qu et al., 2026). Compared with post-activation potentiation (PAP), which emphasizes neural mechanisms, PAPE reflects integrated contributions from elevated body temperature, altered muscle-tendon mechanical properties, improved muscle hydration and ionic balance, and enhanced central drive (Blazevich and Babault, 2019; Prieske et al., 2020). Existing studies confirm that appropriate loading, volume, and recovery intervals following CA using squats, power snatches, or explosive jumps can improve height or power in CMJ or sprint tests, providing a theoretical basis for optimizing pre-game warm-up and pre-training activation (Wilson et al., 2013).

However, PAPE is not guaranteed and is modulated by multiple factors, including CA type, relative intensity and repetitions, movement tempo, rest intervals, individual strength level, training background, and fatigue status (Gołaś et al., 2016; Fischer and Paternoster, 2024). In resistance training settings, insufficient pre-activation load may fail to produce adequate neuromuscular excitation and mechanical stimulus, resulting in weak PAPE. Excessively high load or volume may induce severe metabolic and neuromuscular fatigue that masks or even reverses potential potentiation (Tillin and Bishop, 2009; Seitz and Haff, 2016). Meanwhile, PAPE is highly time-dependent: enhancement typically emerges and peaks within a specific window, then diminishes with prolonged recovery (Sale, 2004). Inconsistencies in testing time points across studies partly explain conflicting findings.

In basketball strength and conditioning, the back squat is a classic resistance exercise for developing lower-limb maximal strength and explosive power, and is widely used as a pre-activation exercise in warm-up (Stojanović et al., 2018). The squat recruits multi-joint muscles of the hip, knee, and ankle extensively, ensures high movement stability, and allows easy intensity adjustment via load manipulation (Lee and Lee, 2018; Straub and Powers, 2024). Research on squat-induced PAPE has focused on effects of relative load on CMJ or SJ height, inter-individual differences in PAPE across strength levels, and influences of sets, reps, and rest intervals on optimal time windows (Fukutani et al., 2014; Si et al., 2026). Overall, some studies report improved CMJ height or power after moderate-to-high load squat CA, while others show no significant PAPE or even mild performance decrement, highlighting the critical role of load selection and matching with recovery intervals.

Most existing research focuses on external performance outcomes such as jump height, with limited attention to neuromuscular activation patterns during jumping. Surface EMG and RMS analysis reveal changes in neuromuscular recruitment strategies across time points (Vigotsky et al., 2018). For SSC-dependent tasks like CMJ—which relies more on tendon elasticity and stretch-reflex feedback than SJ—performance depends on both muscle power and motor coordination of the rapid eccentric-concentric transition (Kubo et al., 1999). Combining jump height with elastic utilization ratio (EUR), a metric quantifying the proportion of jump height derived from stored elastic energy within the SSC, reactive strength index (RSI), an indicator reflecting the efficiency of rapid eccentric-to-concentric transition during countermovement jumps, and EMG measures enables a more comprehensive interpretation of PAPE mechanisms. Furthermore, approach vertical jump (AVJ) and SLJ carry high ecological validity: AVJ approximates in-game driving, lay-up, and dunking by integrating horizontal-to-vertical force transfer, while SLJ reflects horizontal force output and center-of-mass displacement relevant to initial acceleration and sprinting.

Notably, Si et al. (2026) conducted a closely related investigation exploring squat-load mediated PAPE among collegiate female basketball players, yet several critical distinctions differentiate the present study and extend its research scope. First, Si et al. recruited female basketball athletes as participants, whereas the current work focuses exclusively on male collegiate basketball players, addressing the lack of PAPE data specific to male basketball populations. Second, the prior study only assessed raw CMJ and SJ jump height variables, while our research further incorporates elastic utilization ratio (EUR) and reactive strength index (RSI) to comprehensively quantify stretch-shortening cycle efficiency and enrich the evaluation system of explosive performance. Third, Si et al. collected surface EMG signals from only four lower-limb muscles, whereas the present study expanded neuromuscular detection to eight dominant lower-limb muscles, enabling a more complete and holistic analysis of lower-extremity neuromuscular activation coordination during jumping. Collectively, these methodological expansions and sample gender differentiation make the current research a meaningful extension of the existing female-focused PAPE literature in basketball.

Practically, coaches aim to “awaken” the neuromuscular system during pre-game warm-up so that athletes reach near-optimal performance early in competition. However, debates persist regarding optimal squat load for CA and ideal post-CA rest intervals. Systematic comparisons of different squat loads on multiple jumping performances and neuromuscular responses in male collegiate basketball players remain scarce, limiting translation to applied practice.

Accordingly, this study employed a randomized, parallel-group controlled design in male collegiate basketball players to compare acute changes in CMJ, SJ, AVJ, and SLJ at pre-intervention and 4, 8, 12 min post-intervention under 70%, 80%, 90% 1RM parallel back squat CA and a no-load control condition. Ground reaction force and surface EMG of eight dominant lower-limb muscles were synchronously collected during CMJ and SJ to analyze EUR, RSI, and muscle RMS. The objectives were: (1) to clarify the acute time course of jumping performance under different squat loads; (2) to provide actionable guidelines for load selection and optimal rest intervals in warm-up activation; (3) to explain performance differences in PAPE from a neuromuscular activation perspective.

## Methods

### Participants

A 4 (group) × 4 (time point) repeated-measures ANOVA was used. Sample size was estimated using G*Power 3.1 with medium effect size (f = 0.25), α = 0.05, and power = 0.80, yielding a minimum required sample of 24 participants (Tan et al., 2025). Forty male collegiate basketball players were finally enrolled. Inclusion criteria were: (1) national second-class basketball athlete, playing guard or forward; (2) at least 3 years of systematic basketball training; (3) at least one strength training session per week on average in the past year; (4) no musculoskeletal injury in the previous 12 months; (5) no medication or physical therapy affecting athletic performance in the past six months. All participants refrained from strength training and avoided creatine, ergogenic supplements, and stimulants for 48 h before testing. Written informed consent was obtained prior to experimentation. The study was approved by the Ethics Committee of Capital University of Physical Education and Sports (approval number: 2025A168).

### Experimental procedures

This study adopted a randomized, parallel-group controlled design to compare the acute effects of different squat loads on several jump performance indices at multiple post-intervention time points. At enrollment, all participants were randomly assigned to four groups (10 subjects per group). Random allocation was performed using a computer-generated random sequence, with 10 participants assigned to each group. The baseline characteristics of each group are shown in **Table 1**. One-way ANOVA was used to compare baseline characteristics among the four groups, and no significant between-group differences were found for any variable (p > 0.05).

**Table 1:** Baseline characteristics of the participants.

Participant	70% 1RM	80% 1RM	90% 1RM	CG
Age (years)	20.40 ± 0.97	19.90 ± 1.45	20.00 ± 1.70	19.70 ± 1.16
Height (cm)	184.90 ± 3.96	185.20 ± 3.61	185.70 ± 3.20	184.50 ± 5.08
Body Mass (kg)	86.20 ± 5.63	84.30 ± 3.30	82.30± 3.53	85.09 ± 2.77
Training Age (years)	5.20 ± 1.48	5.10 ± 1.10	5.20 ± 0.79	5.00 ± 1.05
1RM Squat (kg)	196.00 ± 13.90	197.55 ± 19.76	190.00 ± 14.53	196.00 ± 12.65

70% 1RM, 70% 1RM squat group; 80% 1RM, 80% 1RM squat group; 90% 1RM, 90% 1RM squat group; CG, no-load control group.

All testing was completed over two separate sessions, with at least 72 hours between sessions. A standardized warm-up procedure was performed before each session. The first session was used to determine 1RM in the back squat. During the second session, participants completed the following in chronological order: pre-intervention jump tests, acute training intervention, and post-intervention jump tests. Post-intervention jump tests were performed at three time points: 4, 8, and 12 min after the intervention. The jump tests were conducted in the following order: CMJ, SJ, AVJ, and standing long jump (SLJ). All tests were closely supervised by experienced researchers and conducted in a sports biomechanics laboratory.

The present study adopted a randomized parallel-group rather than a within-subject crossover design for practical and experimental control reasons. First, each testing session combined eight-channel surface EMG acquisition, force platform measurement, and four sets of jump tests across four time points, requiring approximately 90–120 min of continuous laboratory participation per participant. A crossover design would require each athlete to complete four independent testing visits separated by at least 72 h, which would create prolonged testing cycles, disrupt regular team basketball and strength training routines, and raise participant fatigue and dropout risks. Second, repeated high-load squat stimuli across multiple visits would induce cumulative lower-limb neuromuscular fatigue, altering baseline PAPE responsiveness in subsequent testing days and introducing additional confounding factors. Computer-generated random allocation was used to distribute participants evenly across four groups, and one-way ANOVA confirmed no significant intergroup differences in age, body mass, training experience, or 1RM squat strength at baseline, which partially reduced the confounding impact of individual variability in PAPE susceptibility.

#### 1RM parallel back squat test

Participants performed a high-bar back squat using a 20-kg barbell and calibrated weight plates in a squat rack equipped with safety pins. Two spotters were present during testing. Stance width was self-selected. Squat depth was standardized as the thigh parallel to the floor and was confirmed by two evaluators viewing from the side. Each repetition consisted of a controlled eccentric phase of approximately 2–3 s followed by a maximal-effort concentric phase; bouncing and compensatory movements were not allowed. The warm-up procedure, load progression, rest intervals, and 1RM determination followed the guidelines of the National Strength and Conditioning Association (NSCA) (NSCA-National Strength and Conditioning Association, 2021). The 1RM was determined within a maximum of five formal attempts. A successful attempt had to meet the depth criterion and be completed without external assistance or obvious technical errors.

#### Countermovement jump/squat jump tests

Vertical ground reaction forces were recorded using a force platform (Kistler 9281CA, Switzerland; sampling frequency 1000 Hz). Muscle activity during the tasks was recorded with a wireless EMG system (Delsys Trigno, United States). For the CMJ, participants stood on the force platform with hands on hips and, on a verbal signal, performed a rapid countermovement followed by a maximal vertical jump and landed back on the force platform. During SJ, participants adopted a squat position with knee angle visually verified to be approximately 90° prior to each trial, with hands on hips, and performed a maximal vertical jump without preparatory countermovement before takeoff. After holding this position, they jumped vertically as fast and as high as possible without any preparatory countermovement and landed back on the platform. Each test consisted of three valid trials with approximately 10 s rest between trials. The best performance was used for analysis.

#### Approach vertical jump/standing long jump tests

For the AVJ, an electronic Vertec device (resolution ≥ 0.5 cm) was used to measure AVJ height. Standing reach height was recorded first, and jump height was calculated as the difference between maximal reaching height during the jump and standing reach height. The dominant leg was defined as the preferred takeoff leg during a spontaneous single-leg jump, determined via a pre-test familiarization trial. Participants jumped from their dominant leg and touched the vane with the contralateral hand. The stepping pattern was standardized to three consecutive steps (dominant leg–non-dominant leg–dominant leg), while the approach distance was self-selected by participants to accommodate individual acceleration habits, ensuring consistent movement structure across trials. Each participant completed two valid trials with approximately 10 s rest between trials. If the difference between the two trials exceeded 0.5 cm, a third trial was performed. The best performance was used for analysis. The SLJ was performed indoors on a wooden floor. Jump distance was measured with a tape measure and recorded to the nearest 1 cm.

#### Training intervention

Participants were classified into experimental and control groups according to whether they received the resistance intervention. The experimental groups (70% 1RM, 80% 1RM, and 90% 1RM) performed parallel back squats with loads corresponding to 70%, 80%, and 90% of individual 1RM, respectively, for 3 sets of 4 repetitions, with 90 s rest between sets. Squat depth was standardized as thigh parallel to the floor. The control group did not receive any loading stimulus and remained seated for a standardized 10 min of passive quiet rest after the pre-test, matching the total approximate time required for the squat conditioning activity in experimental groups (3 sets × 4 repetitions with 90 s inter-set rest, total CA duration ~10 min).

### Data processing

#### Ground reaction force data

Three-dimensional ground reaction forces from the force platform were low-pass filtered at 30 Hz using a fourth-order Butterworth filter for subsequent analysis. For CMJ and SJ, the time when the vertical ground reaction force (F_y_) first dropped below 10 N after the start of the trial during take-off was defined as take-off (t_1_), and the time when F_y_ first exceeded 10 N after t_1_ was defined as landing (t_2_). For the CMJ, the onset of the propulsive phase was designated as t_0_, defined as the inflection point where vertical ground reaction force (Fy) shifts from decreasing to increasing after the start of each trial.

#### EMG data

EMG data were segmented according to t_0_ and t_1_. For the present EMG analysis, RMS values were calculated from CMJ trials only. SJ trials were used for jump-performance outcomes but were not included in the tabulated EMG analysis.The EMG system operated at an EMG sampling frequency of 2000 Hz; the system’s built-in pre-amplifier gain was set to 1000×, input impedance > 100 MΩ, and common mode rejection ratio (CMRR) > 80 dB at 50 Hz.

**Table 2:** Muscle RMS results.

Muscle	Group	PRE	POST-4	POST-8	POST-12
RF	70% 1RM	46.37 ± 1.69	50.17 ± 1.89	50.57 ± 2.61	51.21 ± 1.64
	80% 1RM	47.55 ± 1.79	50.71 ± 2.41	52.62 ± 0.89	53.55 ± 1.55
	90% 1RM	47.12 ± 2.50	44.99 ± 2.20	48.58 ± 1.74	49.32 ± 1.64
	CG	46.93 ± 2.11	47.18 ± 1.56	47.98 ± 2.37	48.86 ± 2.30
VL	70% 1RM	35.17 ± 3.43	36.31 ± 4.26	36.34 ± 4.89	39.02 ± 4.25
	80% 1RM	35.79 ± 2.22	36.27 ± 3.26	37.07 ± 4.30	36.11 ± 3.34
	90% 1RM	36.32 ± 3.00	34.19 ± 5.21	34.90 ± 3.31	37.13 ± 5.31
	CG	38.35 ± 2.24	36.57 ± 3.53	37.42 ± 5.20	38.11 ± 4.82
VM	70% 1RM	40.18 ± 2.62	40.11 ± 3.10	39.28 ± 2.91	40.69 ± 3.97
	80% 1RM	40.15 ± 4.80	39.72 ± 4.41	38.30 ± 1.98	40.80 ± 5.76
	90% 1RM	39.75 ± 4.59	40.87 ± 4.09	40.29 ± 2.74	40.31 ± 4.54
	CG	42.27 ± 3.51	40.76 ± 4.20	41.47 ± 3.35	42.45 ± 4.80
TA	70% 1RM	48.69 ± 4.82	48.98 ± 3.63	49.04 ± 3.30	45.35 ± 4.52
	80% 1RM	47.45 ± 5.48	48.94 ± 6.83	46.51 ± 6.04	43.85 ± 2.52
	90% 1RM	48.08 ± 5.36	46.14 ± 6.96	49.21 ± 3.42	47.43 ± 4.34
	CG	48.15 ± 5.13	48.95 ± 3.17	51.81 ± 6.03	49.38 ± 5.00
GM	70% 1RM	21.42 ± 2.83	20.54 ± 2.93	21.17 ± 2.52	20.43 ± 2.34
	80% 1RM	19.22 ± 1.05	18.90 ± 3.61	20.48 ± 2.92	19.97 ± 2.61
	90% 1RM	20.06 ± 3.01	20.04 ± 1.87	20.98 ± 1.88	20.26 ± 2.92
	CG	19.38 ± 3.78	19.18 ± 3.52	21.17 ± 3.73	19.33 ± 2.59
BF	70% 1RM	38.18 ± 2.37	40.97 ± 1.65	42.85 ± 3.05	41.88 ± 2.39
	80% 1RM	39.64 ± 2.55	41.74 ± 2.74	43.31 ± 3.36	43.38 ± 1.77
	90% 1RM	38.70 ± 2.37	37.13 ± 2.82	40.22 ± 2.08	40.46 ± 1.90
	CG	37.91 ± 2.30	37.58 ± 2.55	38.07 ± 3.14	37.62 ± 1.78
ST	70% 1RM	41.67 ± 2.39	39.58 ± 3.35	37.05 ± 1.71	37.66 ± 3.54
	80% 1RM	39.69 ± 5.57	39.12 ± 5.41	39.50 ± 3.22	41.37 ± 3.04
	90% 1RM	39.60 ± 3.55	42.41 ± 6.63	39.41 ± 2.96	38.46 ± 3.84
	CG	39.79 ± 4.56	41.28 ± 3.02	38.80 ± 4.60	38.30 ± 2.63
LG	70% 1RM	50.72 ± 4.35	54.38 ± 3.77	56.04 ± 2.91	55.71 ± 4.58
	80% 1RM	49.15 ± 3.65	54.56 ± 3.52	55.33 ± 4.48	56.98 ± 3.77
	90% 1RM	50.84 ± 3.15	50.19 ± 4.86	54.78 ± 2.70	56.04 ± 2.91
	CG	50.27 ± 4.58	50.60 ± 2.71	50.75 ± 4.51	51.07 ± 3.67

Values are presented as mean ± SD; 70% 1RM, 70% 1RM squat group; 80% 1RM, 80% 1RM squat group; 90% 1RM, 90% 1RM squat group; CG, no-load control group; PRE, pre-intervention; POST-4, 4 min post-intervention; POST-8, 8 min post-intervention; POST-12, 12 min post-intervention.

**Figure 1:**
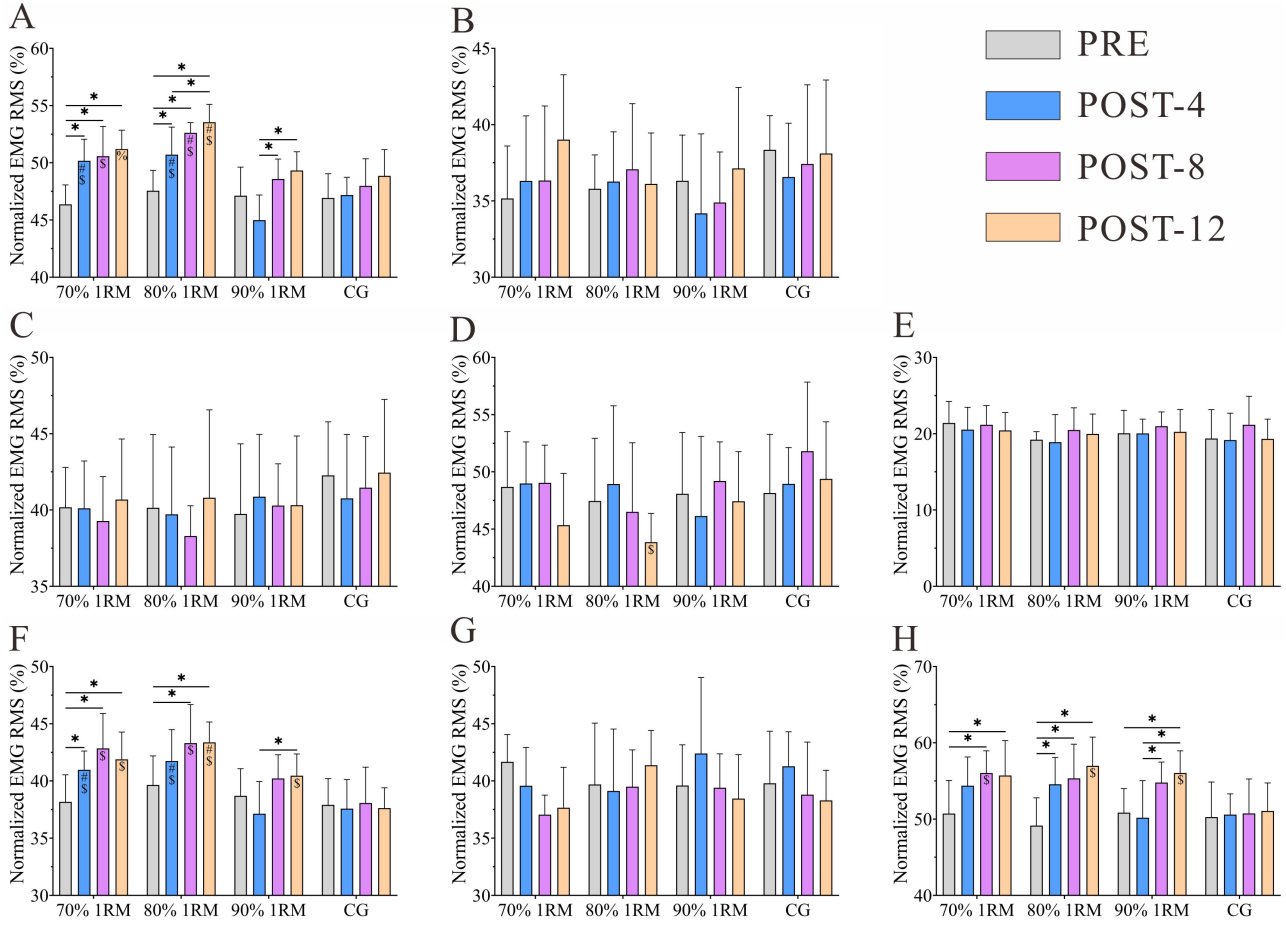
Changes in normalized lower-limb EMG RMS during CMJ after different back-squat conditioning loads. **(A)** Rectus femoris (RF); **(B)** vastus lateralis (VL); **(C)** vastus medialis (VM); **(D)** tibialis anterior (TA); **(E)** gluteus maximus (GM); **(F)** biceps femoris (BF); **(G)** semitendinosus (ST); **(H)** lateral gastrocnemius (LG). The y-axis represents normalized EMG RMS (%). Values are presented as mean ± SD. 70% 1RM, 70% 1RM squat group; 80% 1RM, 80% 1RM squat group; 90% 1RM, 90% 1RM squat group; CG, no-load control group; PRE, pre-intervention; POST-4, POST-8, and POST-12, 4, 8, and 12 min post-intervention, respectively. % indicates p < 0.05 vs. 80% 1RM; # indicates p < 0.05 vs. 90% 1RM; $ indicates p < 0.05 vs. CG; *indicates p < 0.05 for within-group comparisons.

Eight EMG sensors were placed over the dominant lower limb on the rectus femoris (RF), vastus lateralis (VL), vastus medialis (VM), tibialis anterior (TA), gluteus maximus (GM), biceps femoris (BF), semitendinosus (ST), and lateral gastrocnemius (LG). These eight muscles were selected *a priori* to represent the principal superficial muscles of the hip-knee-ankle kinetic chain involved in squat-based CA and vertical jumping. Specifically, RF, VL, and VM were included to characterize quadriceps-mediated knee-extension drive; GM, BF, and ST were included to capture posterior-chain hip extension and hamstring coactivation/knee stabilization; and TA and LG were included to reflect ankle dorsiflexor-plantarflexor control and terminal push-off.

Before electrode placement, the skin was shaved, lightly abraded, and cleaned with alcohol. Disposable Ag/AgCl electrodes were then placed in line with the muscle fiber direction with an inter-electrode distance of approximately 20 mm (Hermens et al., 2000). EMG and force data were synchronized. The segmented EMG signals were band-pass filtered using a fourth-order Butterworth filter (10–400 Hz) (Shi et al., 2023) and then full-wave rectified. A moving RMS window of 0.125 s without overlap was applied to obtain the linear envelope. Finally, for each muscle, EMG amplitudes at all time points were normalized to the peak EMG value obtained from the pre-intervention of the corresponding jump task. The same PRE-derived normalization reference was then applied consistently to pre-intervention, POST-4, POST-8, and POST-12 values. Thus, the normalization denominator remained constant across time points within each muscle, allowing temporal comparisons of normalized RMS values within the same muscle. Because each muscle was normalized to its own PRE peak value, normalized RMS values were not used to compare activation magnitude across different muscles.

A lower band-pass cutoff of 10 Hz was adopted instead of the SENIAM-recommended 20 Hz for two reasons. First, preliminary laboratory tests indicated that a 10 Hz filter effectively captured muscle motion signals. Second, standardized pre-test skin preparation (shaving, abrasion, alcohol cleaning) and secure sensor fixation with elastic tape effectively suppressed motion artifacts, eliminating the need for a higher 20 Hz cutoff. Motion artifact levels were visually inspected for all raw EMG signals prior to signal processing, and any trials with obvious movement noise were discarded and retested.

Two standardized procedures were implemented to minimize and verify signal selectivity and reduce cross-talk between adjacent muscle groups:

For posterior thigh electrodes (BF and ST): After electrode attachment, participants performed isolated voluntary contractions targeting BF (knee flexion with external tibial external rotation) and ST (knee flexion with tibial internal rotation). Real-time EMG waveforms were monitored; electrodes were repositioned if >10% signal amplitude leakage was observed from the non-target hamstring muscle.

For RF: Participants completed isolated hip flexion (minimizing sartorius/tensor fascia latae activation) and isolated knee extension. Real-time EMG amplitude was checked to ensure minimal signal interference from adjacent biarticular muscles.

All electrode positions were re-adjusted and re-validated if substantial cross-talk was detected during isolated contraction checks before formal jump testing.

### Outcome measures

For CMJ and SJ, the outcome variables included CMJ height (cm), SJ height (cm), EUR (%), RSI (m/s), and RMS values of the analyzed muscles during CMJ. The best performance from the formal trials was used for analysis. The calculation formulas for CMJ and SJ were as follows:


$$h=\frac{1}{8}g\times {\left(t_{2}-t_{1}\right)}^{2}$$

where g = 9.8m/s^2^, t_2_ represents the landing time, and t_1_ represents the take-off time.

EUR was computed as:


$$\text{EUR}=\frac{CMJ-SJ}{CMJ}\times 100\%$$

where CMJ and SJ represent the jump heights in the CMJ and squat jump (SJ), respectively. Elastic utilization ratio (EUR) quantifies the contribution of stored elastic energy during the SSC of CMJ.

CMJ and SJ represent the jump heights in the CMJ and SJ, respectively.

RSI was calculated as CMJ jump height divided by the propulsive contraction time (i.e., the time from the onset of the propulsive phase to take-off) (Barker et al., 2018), with the formula expressed as:


$$\text{RSI}=\frac{CMJ}{t_{1}-t_{0}}$$

CMJ refers to countermovement jump height converted to meters, t_1_ represents the take-off time, t_0_ represents the onset of the propulsive phase, and t_1_ - t_0_ represents the propulsive contraction time converted to seconds.

The root mean square (RMS) of the EMG signal was calculated as:


$$RMS=\sqrt{\frac{1}{N}\sum_{i=1}^{N}x_{i}^{2}}$$

where x_i_ represents the EMG value at the i-th sampling point within the time window, each value was normalized to the peak EMG recorded during pre-intervention trials of the CMJ task, and N is the total number of sampling points in the window.

In the AVJ test, the outcome variable was vertical jump height.

In the SLJ test, the outcome variable was jump distance.

### Statistical analysis

All statistical analyses were performed using SPSS 26.0 (IBM Corp., Armonk, NY, USA). The Shapiro–Wilk test was first used to examine the normality of all variables, and the results indicated that all data were normally distributed. All experimental data are presented as mean ± standard deviation (Mean ± SD).

A 4 [group: 70% 1RM squat group (70% 1RM), 80% 1RM squat group (80% 1RM), 90% 1RM squat group (90% 1RM), no-load control group (CG)] × 4 [time: pre-intervention (PRE), 4 min post-intervention (POST-4), 8 min post-intervention (POST-8), 12 min post-intervention (POST-12)] repeated-measures ANOVA was conducted for each outcome variable. Partial eta squared (partial η²) was calculated as a measure of effect size (small effect: η² = 0.01; medium effect: η² = 0.06; large effect: η² = 0.14). When appropriate, Bonferroni-adjusted pairwise comparisons were used for *post hoc* testing. The significance level was set at α = 0.05, and statistical significance was accepted at p < 0.05.

## Results

### Countermovement jump/squat jump

#### Countermovement jump

**Figure 2:**
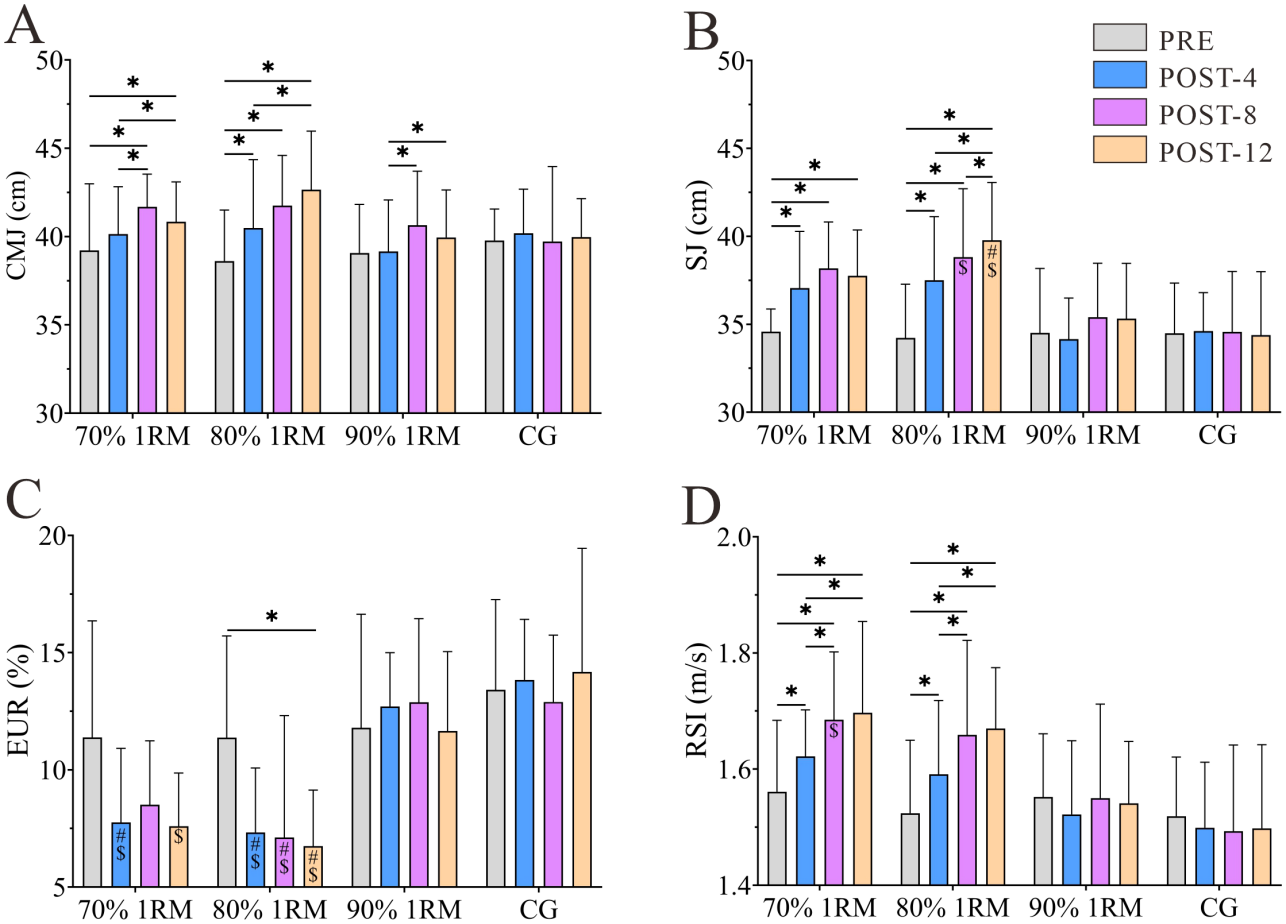
Changes in CMJ, SJ, EUR, and RSI after different back-squat conditioning loads. **(A)** Countermovement jump height (CMJ, cm); **(B)** squat jump height (SJ, cm); **(C)** elastic utilization ratio (EUR, %); **(D)** reactive strength index (RSI, m/s). Values are presented as mean ± SD. 70% 1RM , 70% 1RM squat group; 80% 1RM , 80% 1RM squat group; 90% 1RM , 90% 1RM squat group; CG, no-load control group; PRE, pre-intervention; POST-4, POST-8, and POST-12 , 4, 8, and 12 min post-intervention, respectively. # indicates p < 0.05 vs. 90% 1RM; $ indicates p < 0.05 vs. CG; * indicates p < 0.05 for within-group comparisons.

The CMJ results are presented in **Table 3**. Repeated-measures ANOVA revealed a significant main effect of time (F = 24.392, p < 0.05, η² = 0.404), a non-significant main effect of group (F = 0.366, p > 0.05, η² = 0.030), and a significant time × group interaction (F = 16.239, p < 0.05, η² = 0.575). The multiple comparison results are shown in **Figure 2A**. Within-group comparisons indicated that, in the 70% 1RM group, CMJ height at POST-8 and POST-12 was significantly higher than at PRE and POST-4; in the 80% 1RM group, CMJ height at POST-4, POST-8, and POST-12 was significantly higher than at PRE, and POST-12 was significantly higher than POST-4; in the 90% 1RM group, CMJ height at POST-8 and POST-12 was significantly higher than at POST-4.

**Table 3:** CMJ/SJ test results.

Index	Group	PRE	POST-4	POST-8	POST-12
CMJ	70% 1RM	39.22 ± 3.77	40.14 ± 2.67	41.69 ± 1.84	40.84 ± 2.26
	80% 1RM	38.62 ± 2.89	40.49 ± 3.87	41.75 ± 2.84	42.65 ± 3.32
	90% 1RM	39.07 ± 2.75	39.16 ± 2.92	40.45 ± 3.06	39.95 ± 2.69
	CG	39.78 ± 1.78	40.19 ± 2.50	39.73 ± 4.24	39.97 ± 2.18
SJ	70% 1RM	34.58 ± 1.29	37.06 ± 3.23	38.17 ± 2.65	37.76 ± 2.61
	80% 1RM	34.23 ± 3.05	37.51 ± 3.61	38.81 ± 3.90	39.77 ± 3.29
	90% 1RM	34.51 ± 3.66	34.16 ± 2.33	35.41 ± 3.07	35.32 ± 3.15
	CG	34.49 ± 2.86	34.62 ± 2.19	34.56 ± 3.44	34.38 ± 3.61
EUR	70% 1RM	11.39 ± 4.97	7.76 ± 3.16	8.51 ± 2.73	7.60 ± 2.27
	80% 1RM	11.38 ± 4.34	7.33 ± 2.75	7.12 ± 5.20	6.74 ± 2.39
	90% 1RM	11.79 ± 4.85	12.70 ± 2.30	12.88 ± 3.57	11.65 ± 3.39
	CG	13.42 ± 3.85	13.84 ± 2.58	12.90 ± 2.85	14.17 ± 5.27
RSI	70% 1RM	1.56 ± 0.12	1.62 ± 0.08	1.69 ± 0.12	1.70 ± 0.16
	80% 1RM	1.52 ± 0.13	1.59 ± 0.13	1.66 ± 0.16	1.67 ± 0.10
	90% 1RM	1.55 ± 0.11	1.52 ± 0.13	1.55 ± 0.16	1.54 ± 0.11
	CG	1.52 ± 0.10	1.50 ± 0.11	1.49 ± 0.15	1.50 ± 0.14

Values are presented as mean ± SD; 70% 1RM, 70% 1RM squat group; 80% 1RM, 80% 1RM squat group; 90% 1RM, 90% 1RM squat group; CG, no-load control group; PRE, pre-intervention; POST-4, 4 min post-intervention; POST-8, 8 min post-intervention; POST-12, 12 min post-intervention.

#### Squat jump

The SJ results are presented in **Table 3**. Repeated-measures ANOVA revealed a significant main effect of time (F = 45.684, p < 0.05, η² = 0.559), a non-significant main effect of group (F = 2.647, p > 0.05, η² = 0.181), and a significant time × group interaction (F = 12.674, p < 0.05, η² = 0.514). The multiple comparison results are shown in **Figure 2B**. Between-group comparisons showed that at POST-8, the 80% 1RM group was significantly higher than the control group; at POST-12, the 80% 1RM group was significantly higher than both the 90% 1RM group and the control group. Within-group comparisons indicated that, in the 70% 1RM group, SJ height at POST-4, POST-8, and POST-12 was significantly higher than at PRE; in the 80% 1RM group, SJ height at POST-4, POST-8, and POST-12 was significantly higher than at PRE, and POST-12 was significantly higher than POST-4 and POST-8.

#### Elastic utilization ratio

The EUR results are presented in **Table 3**. Repeated-measures ANOVA revealed a non-significant main effect of time (F = 2.401, p > 0.05, η² = 0.063), a significant main effect of group (F = 17.638, p < 0.05, η² = 0.595), and a non-significant time × group interaction (F = 1.410, p > 0.05, η² = 0.105). The multiple comparison results are shown in **Figure 2C**. Between-group comparisons showed that at POST-4, EUR in the 70% 1RM and 80% 1RM groups was significantly lower than in the 90% 1RM group and the control group; at POST-8, EUR in the 80% 1RM group was significantly lower than in the 90% 1RM group and the control group; at POST-12, EUR in the 70% 1RM and 80% 1RM groups was significantly lower than in the control group, and the 80% 1RM group was significantly lower than the 90% 1RM group. Within-group comparisons showed that, in the 80% 1RM group, EUR at POST-12 was significantly lower than at PRE.

#### Reactive strength index

The RSI results are presented in **Table 3**. Repeated-measures ANOVA revealed a significant main effect of time (F = 23.529, p < 0.05, η² = 0.395), a non-significant main effect of group (F = 2.669, p > 0.05, η² = 0.182), and a significant time × group interaction (F = 11.159, p < 0.05, η² = 0.482). The multiple comparison results are shown in **Figure 2D**. Between-group comparisons showed that at POST-8, the 70% 1RM group was significantly higher than the control group. Within-group comparisons indicated that, in both the 70% 1RM and 80% 1RM groups, RSI at POST-4, POST-8, and POST-12 was significantly higher than at PRE, and RSI at POST-8 and POST-12 was significantly higher than at POST-4.

### Approach vertical jump/standing long jump

#### Approach vertical jump

**Figure 3:**
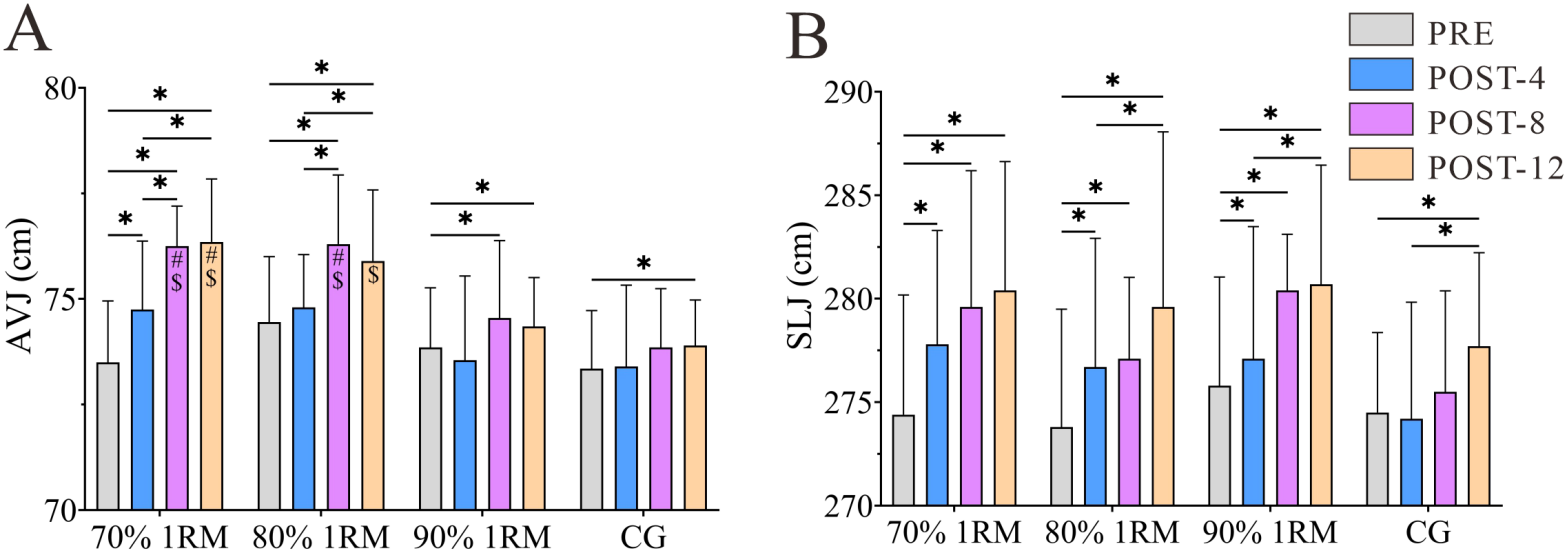
Changes in approach vertical jump and standing long jump after different back-squat conditioning loads. **(A)** Approach vertical jump height (AVJ, cm); **(B)** standing long jump distance (SLJ, cm). Values are presented as mean ± SD. 70% 1RM, 70% 1RM squat group; 80% 1RM, 80% 1RM squat group; 90% 1RM, 90% 1RM squat group; CG, no-load control group; PRE, pre-intervention; POST-4, POST-8, and POST-12, 4, 8, and 12 min post-intervention, respectively. # indicates p < 0.05 vs. 90% 1RM; $ indicates p < 0.05 vs. CG; * indicates p < 0.05 for within-group comparisons.

The AVJ results are presented in **Table 4**. Repeated-measures ANOVA revealed significant main effects of time (F = 80.826, p < 0.05, η² = 0.692) and group (F = 3.456, p < 0.05, η² = 0.224), as well as a significant time × group interaction (F = 10.587, p < 0.05, η² = 0.469). The multiple comparison results are shown in **Figure 3A**. Between-group comparisons showed that at POST-8, the 70% 1RM and 80% 1RM groups were significantly higher than the 90% 1RM group and the control group; at POST-12, the 70% 1RM group was significantly higher than the 90% 1RM group and the control group, and the 80% 1RM group was significantly higher than the control group. Within-group comparisons indicated that, in the 70% 1RM group, AVJ height at POST-4, POST-8, and POST-12 was significantly higher than at PRE, and POST-8 and POST-12 were significantly higher than POST-4; in the 80% 1RM group, AVJ height at POST-8 and POST-12 was significantly higher than at PRE and POST-4; in the 90% 1RM group, AVJ height at POST-8 and POST-12 was significantly higher than at PRE; in the control group, AVJ height at POST-12 was significantly higher than at PRE.

**Table 4:** AVJ/SLJ test results.

Index	Group	PRE	POST-4	POST-8	POST-12
AVJ	70% 1RM	73.50 ± 1.45	74.75 ± 1.62	76.25 ± 0.95	76.35 ± 1.49
	80% 1RM	74.45 ± 1.55	74.80 ± 1.25	76.30 ± 1.64	75.90 ± 1.68
	90% 1RM	73.85 ± 1.42	73.55 ± 1.99	74.55 ± 1.83	74.35 ± 1.16
	CG	73.35 ± 1.38	73.40 ± 1.93	73.85 ± 1.39	73.90 ± 1.07
SLJ	70% 1RM	274.40 ± 5.78	277.80 ± 5.49	279.60 ± 6.59	280.40 ± 6.22
	80% 1RM	273.80 ± 5.69	276.70 ± 6.22	277.10 ± 2.72	279.60 ± 5.76
	90% 1RM	275.80 ± 5.25	277.10 ± 6.38	280.40 ± 2.72	280.70 ± 5.76
	CG	274.50 ± 3.87	274.20 ± 5.63	275.50 ± 4.88	277.70 ± 4.52

Values are presented as mean ± SD; 70% 1RM, 70% 1RM squat group; 80% 1RM, 80% 1RM squat group; 90% 1RM, 90% 1RM squat group; CG, no-load control group; PRE, pre-intervention; POST-4, 4 min post-intervention; POST-8, 8 min post-intervention; POST-12, 12 min post-intervention.

#### Standing long jump

The SLJ results are presented in **Table 4**. Repeated-measures ANOVA revealed a significant main effect of time (F = 42.408, p < 0.05, η² = 0.541), a non-significant main effect of group (F = 0.656, p > 0.05, η² = 0.052), and a significant time × group interaction (F = 2.055, p < 0.05, η² = 0.146). The multiple comparison results are shown in **Figure 3B**. Within-group comparisons indicated that, in the 70% 1RM group, SLJ distance at POST-4, POST-8, and POST-12 was significantly greater than at PRE; in the 80% 1RM group, SLJ distance at POST-4, POST-8, and POST-12 was significantly greater than at PRE, and POST-12 was significantly greater than POST-4; in the 90% 1RM group, SLJ distance at POST-8 and POST-12 was significantly greater than at PRE and POST-4; in the control group, SLJ distance at POST-12 was significantly greater than at PRE and POST-4.

### Muscle RMS

All RMS values in **Table 2** and **Figure 1** represent normalized EMG amplitudes obtained from CMJ trials.

#### Rectus femoris

The RF RMS results are presented in **Table 2**. Repeated-measures ANOVA revealed significant main effects of time (F = 25.579, p < 0.05, η² = 0.415) and group (F = 46.279, p < 0.05, η² = 0.794), as well as a significant time × group interaction (F = 3.704, p < 0.05, η² = 0.236). The multiple comparison results are shown in **Figure 1A**. Between-group comparisons showed that at POST-4, RF RMS in the 70% 1RM and 80% 1RM groups was significantly higher than in the 90% 1RM group and the control group; at POST-8, RF RMS in the 70% 1RM and 80% 1RM groups was significantly higher than in the control group, and the 80% 1RM group was significantly higher than the 90% 1RM group; at POST-12, RF RMS in the 80% 1RM group was significantly higher than in the other three groups, and the 70% 1RM group was significantly higher than the control group. Within-group comparisons indicated that, in the 70% 1RM group, RF RMS at POST-4, POST-8, and POST-12 was significantly higher than at PRE; in the 80% 1RM group, RF RMS at POST-4, POST-8, and POST-12 was significantly higher than at PRE, and POST-12 was significantly higher than POST-4; in the 90% 1RM group, RF RMS at POST-8 and POST-12 was significantly higher than at POST-4.

#### Vastus lateralis

The VL RMS results are presented in **Table 2**. Repeated-measures ANOVA revealed non-significant main effects of time (F = 1.228, p > 0.05, η² = 0.033) and group (F = 2.299, p > 0.05, η² = 0.161), and a non-significant time × group interaction (F = 0.604, p > 0.05, η² = 0.048). The multiple comparison results are shown in **Figure 1B**, and no pairwise between-group differences reached statistical significance.

#### Vastus medialis

The VM RMS results are presented in **Table 2**. Repeated-measures ANOVA revealed non-significant main effects of time (F = 0.722, p > 0.05, η² = 0.020) and group (F = 1.605, p > 0.05, η² = 0.118), and a non-significant time × group interaction (F = 0.287, p > 0.05, η² = 0.023). The multiple comparison results are shown in **Figure 1C**, and no pairwise between-group differences reached statistical significance.

#### Tibialis anterior

The TA RMS results are presented in **Table 2**. Repeated-measures ANOVA revealed a non-significant main effect of time (F = 1.803, p > 0.05, η² = 0.048), a significant main effect of group (F = 3.208, p < 0.05, η² = 0.211), and a non-significant time × group interaction (F = 0.878, p > 0.05, η² = 0.068). The multiple comparison results are shown in **Figure 1D**. Between-group comparisons showed that at POST-12, TA RMS in the 80% 1RM group was significantly lower than in the control group.

#### Gluteus maximus

The GM RMS results are presented in **Table 2**. Repeated-measures ANOVA revealed non-significant main effects of time (F = 1.490, p > 0.05, η² = 0.040) and group (F = 1.706, p > 0.05, η² = 0.124), and a non-significant time × group interaction (F = 0.245, p > 0.05, η² = 0.020). The multiple comparison results are shown in **Figure 1E**, and no pairwise between-group differences reached statistical significance.

#### Biceps femoris

The BF RMS results are presented in **Table 2**. Repeated-measures ANOVA revealed significant main effects of time (F = 9.396, p < 0.05, η² = 0.207) and group (F = 22.500, p < 0.05, η² = 0.652), as well as a significant time × group interaction (F = 2.109, p < 0.05, η² = …). The multiple comparison results are shown in **Figure 1F**. Between-group comparisons showed that at POST-4, BF RMS in the 70% 1RM and 80% 1RM groups was significantly higher than in the 90% 1RM group and the control group; at POST-8, BF RMS in the 70% 1RM and 80% 1RM groups was significantly higher than in the control group; at POST-12, BF RMS in the 70% 1RM, 80% 1RM, and 90% 1RM groups was significantly higher than in the control group, and the 80% 1RM group was significantly higher than the 90% 1RM group. Within-group comparisons indicated that, in the 70% 1RM group, BF RMS at POST-4, POST-8, and POST-12 was significantly higher than at PRE; in the 80% 1RM group, BF RMS at POST-8 and POST-12 was significantly higher than at PRE; in the 90% 1RM group, BF RMS at POST-12 was significantly higher than at POST-4.

#### Semitendinosus

The ST RMS results are presented in **Table 2**. Repeated-measures ANOVA revealed non-significant main effects of time (F = 2.185, p > 0.05, η² = 0.057) and group (F = 0.568, p > 0.05, η² = 0.045), and a non-significant time × group interaction (F = 1.366, p > 0.05, η² = 0.102). The multiple comparison results are shown in **Figure 1G**, and no pairwise between-group differences reached statistical significance.

#### Lateral gastrocnemius

The LG RMS results are presented in **Table 2**. Repeated-measures ANOVA revealed significant main effects of time (F = 14.657, p < 0.05, η² = 0.289) and group (F = 4.684, p < 0.05, η² = 0.281), as well as a significant time × group interaction (F = 2.293, p < 0.05, η² = 0.160). The multiple comparison results are shown in **Figure 1H**. Between-group comparisons showed that at POST-8, LG RMS in the 70% 1RM group was significantly higher than in the control group; at POST-12, LG RMS in the 80% 1RM and 90% 1RM groups was significantly higher than in the control group. Within-group comparisons indicated that, in the 70% 1RM group, LG RMS at POST-8 and POST-12 was significantly higher than at PRE; in the 80% 1RM group, LG RMS at POST-4, POST-8, and POST-12 was significantly higher than at PRE; in the 90% 1RM group, LG RMS at POST-12 was significantly higher than at PRE, and LG RMS at POST-8 and POST-12 was significantly higher than at POST-4.

### *Post-hoc* power analysis

*Post-hoc* power was examined for observed effects with Cohen’s f values greater than 0.99 by converting η^2^ values to Cohen’s f using f = $\sqrt{{\text{η}}^{2}÷(1-{\text{η}}^{2})}$.

The effects meeting this criterion were the CMJ time × group interaction (η^2^ = 0.575, f = 1.16), the SJ main effect of time (η^2^ = 0.559, f = 1.13), the SJ time × group interaction (η^2^ = 0.514, f = 1.03), the EUR main effect of group (η^2^ = 0.595, f = 1.21), the AVJ main effect of time (η^2^ = 0.692, f = 1.50), the SLJ main effect of time (η^2^ = 0.541, f = 1.09), the RF RMS main effect of group (η^2^ = 0.794, f = 1.96), and the BF RMS main effect of group (η^2^ = 0.652, f = 1.37). For these effects, the observed *post-hoc* power exceeded 0.99 at alpha = 0.05. The discrepancy between the *a priori* assumed medium effect size (f = 0.25) and these larger observed effects likely reflects the conservative planning assumption used before data collection and the pronounced load-specific responses observed in this sample.

## Discussion

This study examined the acute changes in CMJ, SJ, AVJ, standing long jump (SLJ), and surface EMG activity of eight lower-limb muscles in male collegiate basketball players following parallel back squat conditioning activities (CAs) at 70%, 80%, and 90% of 1RM. The aim was to characterize the PAPE response of basketball-related jump performance and to explore the underlying neuromuscular mechanisms. The findings suggest that the effects of squat-based CA on jump performance are both load-specific and time-dependent. Moderate-to-high loads (70%–80% 1RM) appeared to enhance jump performance mainly by modifying neuromuscular recruitment patterns, improving the efficiency of stretch–shortening cycle (SSC) utilization, and increasing concentric force output, whereas the potentiating effect of 90% 1RM was likely constrained by accumulated fatigue. The following sections discuss these findings from the perspectives of vertical explosive power, sport-specific jump performance, and neuromuscular regulation.

### Effects of different squat loads on CMJ and SJ

CMJ and SJ are core indicators of lower-limb vertical explosive power, with CMJ relying primarily on SSC mechanisms and SJ on pure concentric contraction (Bobbert et al., 1996; Van Hooren and Zolotarjova, 2017). Their distinct mechanical bases lead to different response patterns to CA load, which provides an important window into the mechanisms of PAPE. In this study, CMJ height showed a significant time × group interaction. Both the 70% 1RM and 80% 1RM squat CAs led to significant improvements, with the greatest and most prolonged enhancement observed in the 80% 1RM group, which peaked at 12 min post-CA and remained significantly higher than baseline. In the 70% 1RM group, CMJ performance peaked at 8 min and then slightly declined, showing a “rapid onset with mild decline” pattern. In contrast, the 90% 1RM group showed only a small increase at 8 min post-CA and no clear change at 4 min, with an overall potentiating effect markedly weaker than that of the moderate-load groups. The different timing between the 70% and 80% 1RM groups may reflect a load-dependent balance between neuromuscular excitation and residual fatigue. The 70% 1RM condition may have produced a lighter fatigue burden and therefore an earlier potentiation peak at POST-8, whereas the 80% 1RM condition may have provided a stronger conditioning stimulus that required a slightly longer recovery period before its full effect emerged at POST-12.

These results align well with current PAPE frameworks, which posit that CA influences explosive performance through the interaction of at least two competing processes. On the one hand, neuromuscular activation is enhanced: resistance exercise with at least moderate loads may increase central drive, recruit more high-threshold motor units, and elevate discharge rates, thereby improving rapid force production. On the other hand, fatigue can counteract these benefits: when loads approach 1RM, increased depletion of energy substrates, accumulation of metabolites, and heightened inhibitory influences can mask or even offset the gains in neural activation (Chiu et al., 2004; Tillin and Bishop, 2009; Harries et al., 2018). The absence of early performance enhancement at 4 min in the 90% 1RM group in this study likely reflects transient dominance of fatigue at very high loads, with only a modest potentiating effect emerging once fatigue had partially dissipated at 8 min. By contrast, 70% and 80% 1RM appeared to strike a more favorable balance between activation and fatigue, providing sufficient neuromuscular priming without excessive fatigue and thus yielding more stable PAPE responses.

As a purely concentric movement without countermovement or elastic energy storage, SJ may be particularly sensitive to changes in CA load. Here, SJ height showed significant main and interaction effects of time, with the 80% 1RM group displaying larger gains than in CMJ, gradually increasing over time and being significantly higher than both the 90% 1RM group and the control group at 12 min post-CA. The 70% 1RM group showed significant improvements as early as 4 min, peaking at 8 min, whereas the 90% 1RM group did not exhibit statistically significant gains at any time point. These patterns indicate that concentric-dominant explosive performance may rely more heavily on the neural drive enhancements induced by CA. When CA load is moderate, contractile efficiency and maximal concentric force can be elevated concurrently, whereas excessive fatigue at very high loads may suppress concentric force production and limit performance improvements (Andrews et al., 2016).

These findings are partly consistent with those of Si et al. (2026), who examined PAPE induced by 70%, 80%, and 90% 1RM parallel back-squat CAs in collegiate female basketball players using the same 4-, 8-, and 12-min post-CA observation window. Both studies indicate that squat-induced PAPE is load- and time-dependent and that an 80% 1RM load combined with an 8–12 min recovery period may be a practical strategy for enhancing basketball-related jump performance. However, whereas Si et al. reported that loads ≥80% 1RM reliably elicited PAPE and that 90% 1RM produced a delayed rectus femoris activation peak and later CMJ power peak, the present male sample showed more stable CMJ/SJ improvements after 70%–80% 1RM and a weaker vertical-jump response after 90% 1RM. This discrepancy may be related to sex-specific neuromuscular characteristics, differences in training background, and protocol differences such as squat volume (3 × 3 vs. 3 × 4 repetitions) and jump-task selection.

EUR and RSI further illuminate the modulation of SSC behavior by CA. In this study, EUR in the 70% and 80% 1RM groups was significantly lower than in the 90% 1RM and control groups, with a continued decline over time in the 80% 1RM condition. Because EUR represents the relative difference between CMJ and SJ heights, a lower value should be interpreted cautiously. One possible interpretation is that squat-based CA produced a proportionally greater improvement in SJ performance and concentric force production than in CMJ performance, thereby narrowing the CMJ-SJ difference. Alternatively, a lower EUR may also indicate a reduced relative contribution from SSC-related elastic energy storage or stretch-reflex mechanisms during CMJ (Samozino et al., 2008). Therefore, the EUR results alone cannot be taken as direct evidence of improved neuromuscular efficiency; rather, they should be interpreted together with the increases in jump height and RSI. In the present study, CMJ and SJ heights were elevated following conditioning in all experimental groups, and RSI increased significantly only in the 70% and 80% 1RM groups, suggesting that moderate-load CA was indeed capable of enhancing the rapid eccentric-to-concentric transition. This combined pattern may reflect a shift toward stronger concentric force production with maintained or improved reactive performance, which is compatible with basketball-specific demands such as repeated jumping and rapid second jumps.

Taken together, the CMJ and SJ results suggest that the 70% and 80% 1RM squat CAs produced more consistent vertical jump responses than the 90% 1RM condition. The 80% 1RM condition showed a descriptively favorable overall profile across CMJ, SJ, RSI, and selected EMG outcomes; however, because no direct statistically significant CMJ difference was observed between the 70% and 80% 1RM groups, this pattern should be interpreted descriptively rather than as evidence of clear statistical superiority. The 70% 1RM condition appeared to act more quickly with lower mechanical stress, which may be useful in rapid activation scenarios. In contrast, a 90% 1RM squat CA, likely due to the stronger fatigue component, does not appear suitable as a load choice when the goal is to acutely enhance vertical explosive performance.

### Effects of different squat loads on AVJ and SLJ

AVJ and SLJ are more closely aligned with basketball-specific game actions, reflecting approach jump capacity and horizontal explosive power, respectively. These abilities are directly linked to key skills such as lay-ups, shot blocking, initial acceleration, and change-of-direction, and thus represent critical components of sport-specific fitness in basketball (Tai et al., 2019; Theodorou et al., 2022). In this study, AVJ and SLJ showed distinct response patterns to CA load, which may reflect the task-specific nature of PAPE under different force-production modes.

AVJ, characterized by a single-leg approach jump, combines horizontal-to-vertical velocity conversion, vertical force generation, and complex coordination control. Performance in this task places high demands on both neuromuscular activation and technical stability. Here, AVJ height showed significant main effects of time and group and a significant interaction. The 70% and 80% 1RM groups peaked at 8 min post-CA, with values significantly higher than both the 90% 1RM group and the control group, and remained elevated at 12 min. The 90% 1RM group showed only modest gains between 8 and 12 min, with effect sizes clearly smaller than those observed with moderate loads. These findings imply that improvement in approach jump performance may depend on an appropriate level of neuromuscular activation in combination with preserved movement coordination. Squat CAs at moderate loads may enhance central excitability, refine the timing of leg extension, and improve the efficiency of horizontal-to-vertical force transfer while avoiding fatigue-related disruption of the coordination between approach and take-off. In contrast, the fatigue induced by 90% 1RM may reduce leg extension speed and joint stability, thereby disturbing the movement pattern of the approach jump and limiting performance gains (Sun et al., 2019).

In contrast to AVJ, SLJ exhibited a somewhat different pattern in response to high-load CA. In this study, all three loading conditions led to significant increases in SLJ distance; at POST-8, the 90% 1RM group showed the highest absolute SLJ value, whereas the largest within-group gain from PRE to POST-8 occurred in the 70% 1RM group. The 70% and 80% 1RM groups also improved progressively and peaked at 12 min. This divergence may be explained by the biomechanical characteristics of SLJ: standing long jump is dominated by horizontal force production, involves a larger number of contributing muscles, a longer force application time, and a force pattern that is more similar to squat-based hip–knee–ankle extension. High-load squats may provide a stronger mechanical stimulus to hip and knee extensors, thereby increasing overall propulsive force and forward displacement of the center of mass (Mackala et al., 2013). Moreover, the demands for very rapid force production are generally lower in horizontal jumping than in vertical jumping, which may reduce the extent to which fatigue masks performance in SLJ (Knihs et al., 2022). This may also explain why the 90% 1RM group showed clear SLJ improvement at POST-8 and POST-12 but only limited improvement in AVJ at the same time points. AVJ requires approach-to-take-off coordination, rapid vertical impulse generation, and single-leg technical stability; these features may be more vulnerable to residual fatigue after very heavy squat loading. By contrast, SLJ allows a longer bilateral propulsive phase and may benefit more directly from the high-load stimulus to horizontal hip-knee extension. Under these conditions, even a 90% 1RM CA may still permit noticeable improvement in horizontal explosive performance while providing less benefit for more technically demanding vertical approach jumping. At the same time, the control group also showed a significant increase in SLJ at POST-12 despite receiving no CA. This finding suggests that repeated testing, familiarization, or a general warm-up/passive recovery effect may have contributed to some improvement in SLJ performance. Therefore, PAPE-related changes in SLJ should be interpreted with reference to the control condition, and the possibility of a learning or repeated-trial effect should be considered when comparing changes across groups. The relatively smooth and less fluctuating improvements observed with 70% and 80% 1RM may be more suitable when practitioners aim to concurrently enhance both vertical and horizontal explosive power.

From a basketball-specific perspective, players at the beginning of a game often need to perform both vertical actions (e.g., rebounds, blocks) and horizontal actions (e.g., initial sprinting, penetration). Squat CAs at 70%–80% 1RM in the present study appeared to concurrently enhance AVJ and SLJ performance, with the most favorable window emerging around 8–12 min after CA, which aligns well with the early-phase demands of a competitive game. These findings complement previous work that has focused predominantly on vertical jumps and paid less attention to basketball-specific jump tasks. They support the notion that squat-based CA can positively influence game-related jump performance and that load selection may need to be aligned with the specific force-production patterns required by the sport.

### Effects of squat-based CA on neuromuscular activation of lower-limb muscles

Neuromuscular activation patterns are central to the mechanisms of PAPE, but surface EMG amplitude should be interpreted cautiously. In this study, the EMG RMS values were derived from CMJ trials. CMJ was selected for the tabulated EMG analysis because it includes a rapid eccentric-to-concentric transition and SSC contribution, whereas SJ is primarily concentric; these mechanical differences can produce distinct neuromuscular activation patterns during jumping (Bobbert et al., 1996; Padulo et al., 2013). Therefore, focusing the EMG analysis on CMJ allowed the neuromuscular data to be interpreted in relation to the SSC-dependent jump task most relevant to PAPE mechanisms, while SJ was retained as an external performance outcome for evaluating concentric force production. Surface EMG RMS values were collected simultaneously from eight major lower-limb muscles--RF, VL, VM, TA, GM, BF, ST, and LG. Importantly, Olsen et al. emphasized that changes in sEMG amplitude are not consistent mechanistic markers of PAP/PAPE, and that performance improvements may occur despite unchanged or decreased EMG amplitude (Olsen et al., 2025). Accordingly, the RMS findings in the present study should be regarded as supportive descriptive evidence of task-specific neuromuscular responses rather than direct proof of the physiological mechanism underlying PAPE.

RF RMS showed significant main effects of time and group and a significant interaction, whereas VL and VM did not show comparable post-CA changes. Although RF, VL, and VM are all quadriceps muscles innervated by the femoral nerve and contribute to knee extension, RF differs functionally because it is biarticular: it contributes to knee extension while also crossing the hip joint and participating in hip flexion and control of hip-thigh position during the countermovement and take-off phases. The selective RF response may therefore reflect greater involvement of biarticular hip-knee coordination during CMJ after squat CA rather than a uniform increase in quadriceps activation. RMS values in the 70% and 80% 1RM groups increased continuously after CA, with the 80% 1RM group being significantly higher than all other groups at 12 min. By contrast, the 90% 1RM group displayed a decrease in RF activation at 4 min, followed by a gradual recovery. This pattern may indicate a delayed balance between neural facilitation and fatigue in RF. However, this interpretation should be made cautiously, because RF surface EMG is sensitive to electrode placement and signal quality due to its superficial location and proximity to adjacent thigh muscles. Although electrode placement followed standardized SENIAM procedures (Hermens et al., 2000), residual placement-related variability cannot be excluded.

BF, which contributes to hip extension and knee joint stabilization, showed an activation pattern similar to that of RF, whereas ST did not show significant post-CA changes. This BF-ST dissociation may reflect task-specific recruitment of the lateral hamstrings during hip extension and knee stabilization in CMJ, while the medial hamstrings may have contributed more consistently across time points without a detectable RMS increase. However, BF and ST are anatomically adjacent, and surface EMG recordings from these muscles can be affected by cross-talk and electrode selectivity. Therefore, the stronger BF response should be interpreted as a cautious indication of lateral hamstring involvement rather than definitive evidence that ST was not functionally engaged. RMS values in the 70% and 80% 1RM groups were consistently higher than in the control group at all post-CA time points, and in the 90% 1RM group BF RMS was also higher than in the control group at 12 min. These findings suggest that squat-based CAs may refine the hip-knee extension synergy along the kinetic chain, but the muscle-specific interpretation remains limited by the spatial resolution of surface EMG.

LG is a key contributor to ankle plantarflexion and elastic energy storage, and its activation plays an important role in SSC efficiency (Komi and Bosco, 1978). In this study, LG RMS increased significantly after CA; the 70% 1RM group showed higher values than the control group at 8–12 min, and the 80% and 90% 1RM groups were higher than the control group at 12 min. This pattern suggests that squat-based CA may enhance the activation of plantarflexor muscles, thereby improving terminal push-off force and the utilization of tendon elastic energy. These EMG findings appear to be consistent with the observed increases in RSI in the moderate-load groups and together support the interpretation that partial optimization of SSC behavior may be one component of the PAPE response (Ishikawa et al., 2005; Gago et al., 2017).

For the remaining muscles (VL, VM, GM, ST), no significant interaction effects were observed, and pairwise comparisons did not reveal clear between-group differences. This may indicate that these muscles function primarily to stabilize joints and maintain movement control during jumping, rather than being the primary loci of CA-induced performance gains. TA, as an antagonist to plantarflexion,showed lower RMS at POST-12 in the 80% 1RM group than in the control group, which may reflect relatively greater dominance of plantarflexor activation and enhanced antagonist inhibition after CA, consistent with more efficient coordination within agonist–antagonist pairs. However, this finding was based on a single significant comparison at one time point. Because a plantarflexor-dorsiflexor co-activation index and detailed timing analysis were not calculated, the interpretation that reduced TA RMS reflects enhanced antagonist inhibition should be regarded as a hypothesis rather than a firm conclusion.

Overall, the present findings suggest that the neuromuscular effects induced by squat-based pre-activation were not characterized by a generalized increase in activation across all muscle groups. Instead, they reflected a selective enhancement of the RF, BF, and LG. Such a targeted activation pattern may support more efficient explosive force production while avoiding unnecessary energy expenditure, which appears well aligned with the intermittent, high-intensity, multi-joint demands of basketball. This neuromuscular profile may help provide a physiological basis for the observed improvements in jump performance following appropriately loaded squat CAs.

### Limitations

Several limitations should be considered when interpreting the present findings.

First, the randomized parallel between-subject design may be less rigorous than the within-subject crossover design commonly recommended in PAPE research (Wilson et al., 2013; Seitz and Haff, 2016). Although baseline characteristics were comparable among groups, unmeasured individual differences in PAPE responsiveness may still have influenced the load-specific outcomes. Future studies should use crossover designs to better control for inter-individual variability and confirm the present load-response findings.

Second, the sample included only highly trained male collegiate basketball guards and forwards, with center players excluded. Given the positional differences in body morphology, lower-limb strength, jumping mechanics, and movement demands, the identified optimal squat load and 8–12 min potentiation window should not be directly generalized to center players, female athletes, other sports, or lower-trained populations. Moreover, because CA-induced neural facilitation may differ according to training status and may be limited in highly trained athletes, as suggested by Fischer et al. (2024), the present EMG findings should be interpreted as specific to this trained basketball cohort and should not be directly generalized to less-trained individuals (Fischer and Paternoster, 2024).

Third, only the parallel back squat was used as the conditioning activity. Therefore, the findings cannot determine whether similar PAPE responses would occur after other pre-activation exercises, such as front squats, plyometric exercises, or heavy hip thrusts.

Fourth, all tests were conducted in a controlled laboratory environment, which did not fully reproduce the fatigue, psychological stress, and tactical demands of real basketball competition. The practical effectiveness of PAPE under training or in-game conditions requires further verification.

Finally, several EMG-related limitations should be acknowledged. The normalization method allowed within-muscle temporal comparisons but may not fully support direct comparisons across muscles, especially if the reference value contained noise or atypical activation. In addition, although standardized electrode placement procedures were followed, cross-talk may still have occurred between adjacent muscles, particularly BF-ST and RF with neighboring anterior-thigh muscles.

## Conclusion

This study compared the acute effects of loaded parallel back squat conditioning activities on jump performance and lower-limb EMG activity in male collegiate basketball players. The findings suggest that moderately loaded squats can elicit a clear PAPE effect within a short recovery period, with most jump outcomes peaking approximately 8–12 min after the conditioning activity. Compared with 90% 1RM, loads of 70%–80% 1RM produced more consistent improvements in CMJ, SJ, AVJ, RSI, and normalized RMS of RF, BF, and LG, indicating a more favorable balance between potentiation and fatigue. Overall, the 80% 1RM condition showed a descriptively better response pattern than 70% 1RM when jump and EMG outcomes were considered together; however, this should not be interpreted as statistically superior and requires confirmation in larger and more diverse samples.

Practically, the 80% 1RM group improved CMJ height by approximately 4 cm from PRE to POST-12, which may be meaningful for basketball actions such as rebounding, shot blocking, and repeated jumping. However, because typical error and smallest worthwhile change were not calculated for this sample, the competitive relevance of this improvement should be interpreted cautiously.

## Data Availability

The original contributions presented in the study are included in the article/**Supplementary Material**. Further inquiries can be directed to the corresponding author.
